# Hydrostatic low‐volume enemas in infants with birth weight ≤1000 g or gestational age ≤28 weeks: A controlled interventional study

**DOI:** 10.1002/jpn3.70055

**Published:** 2025-05-08

**Authors:** Tabea Stock, Anne‐Marie Kamp, Markus Waitz, Teresa Riedl‐Seifert, Andreas C. Jenke

**Affiliations:** ^1^ Children's Hospital Kassel, Klinikum Kassel Germany; ^2^ Centre for Biomedical Education and Research (ZBAF) Witten/Herdecke University Kassel Germany

**Keywords:** necrotizing enterocolitis, premature infant, rectal enema

## Abstract

**Objective:**

This study evaluated the safety and efficacy of standardized minimally invasive hydrostatic low‐volume saline enemas in infants with a birth weight ≤1000 g or gestational age ≤28 weeks and delayed meconium passage.

**Methods:**

Conducted at the Neonatology Department of Klinikum Kassel, Germany, this monocentric controlled interventional study included a historic control group and a prospective intervention group. Infants born between January 2019 and October 2022 were included. The control group received manual enemas using varied techniques, while the intervention group underwent standardized hydrostatic low‐volume saline enemas at predefined intervals. Key outcomes assessed included gastrointestinal complications (necrotizing enterocolitis [NEC], focal intestinal perforation [FIP], and meconium plug syndrome [MPS]), morbidity, mortality, stool and feeding parameters, and staff evaluations.

**Results:**

A total of 42 infants were included in the control group and 74 in the intervention group. NEC incidence was lower in the intervention group (4.1%) compared to the control group (9.5%), as was the rate of FIP (2.7% vs. 7.1%). Morbidity showed a decreasing trend in the intervention group (6.8% vs. 16.7%), and the combined morbidity and mortality rate was significantly lower (6.8% vs. 19.1%). Despite reduced stool frequency, enteral feeding tolerance improved in the intervention group.

**Conclusion:**

An unstandardized approach to rectal interventions may increase the need for surgical interventions, whereas standardized hydrostatic low‐volume saline enemas are a safe and effective alternative to conventional rectal interventions, offering improved comfort and potentially reducing intestinal morbidity in infants with a birth weight ≤1000 g or gestational age ≤28 weeks.

**Trial Identification Number:**

DRKS00024191 (https://drks.de/search/de/trial/DRKS00024191).

## Abstract (highlights)

**What is Known:**

- Rectal interventions are commonly used in neonatology to aid meconium passage in extremely low birth weight (ELBW) infants.
- There is a lack of robust efficacy and safety data supporting these practices.

**What is New:**

- A standardized protocol using hydrostatic low‐volume saline enemas at pre‐set intervals is shown to be safe.
- The protocol is associated with a trend toward reduced need for surgical management in ELBW infants.
- Compared to unstandardized methods, hydrostatic low‐volume saline enemas offer a more consistent, gentler, and controlled approach.

## Graphical abstract

Standardized hydrostatic enemas improve the intestinal outcome in premature infants.

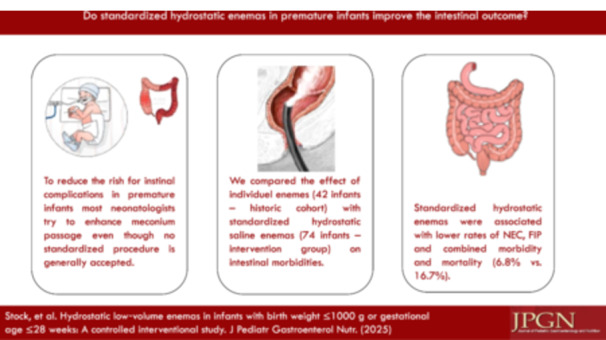

## 1 INTRODUCTION

Infants born with a birth weight below 1000 g are known as extremely low birth weight (ELBW) infants.^1^ In this patient group, the organs, including the gastrointestinal (GI) tract, are immature, and many patients have a delayed meconium passage (MP).^2^ Meanwhile, mature infants pass their first stool within the first 24 h of life,^3^ this is only valid for 44% of infants born under 30 weeks of gestation. Further, meconium evacuation is completed at about 7.8 days (SD: 2.5) and may last up to 22 days.^4^, ^5^ Moreover, ELBW infants are prone to severe GI disorders such as necrotizing enterocolitis (NEC),^6^ focal intestinal perforation (FIP),^7^ and meconium plug syndrome (MPS).^8^ To reduce the risk of these complications, most neonatologists try to enhance the MP by rectal interventions of varying techniques, materials, and frequencies. Enemas are more commonly applied than suppositories.^9^ Interestingly, evidence of rectal interventions' safety and efficacy, particularly concerning reducing the risk of NEC, FIP, and MPS rates, is unclear. On the other hand, it has been hypothesized that repeated episodes of hypoxia caused by rectal interventions may damage the intestinal wall and thereby contribute to GI complications.^10^ In 2016, the systematic review of Kamphorst et al.^11^ concluded that suppositories did not enhance the time until full meconium evacuation was reached, and evidence for the effectiveness of enemas was unclear. Similarly, Deshmukh et al.^12^ did not find a link between NEC risk and rectal interventions in their systematic review and meta‐analysis. A meta‐analysis by Livingston et al.,^13^ identified an earlier stooling initiation (2 vs. 4 days) when using rectal interventions; however, it was associated with a trend towards a slightly increased NEC risk (RR = 2.2, 95% CI: 0.76–9.81, *p* = 0.13). The same research group performed another meta‐analysis in 2022, in which this trend was no longer evident (RR = 1.30; *p* = 0.62), and a reduction in the time of full meconium evacuation was seen (mean: 1.5 days; 95% CI: 3.0–0.01; *p* = 0.05). However, this had no clinical impact since NEC and mortality rates were not altered.^14^

Another challenge in the management of ELBW infants remains enteral food tolerance. Again, it is believed that a quicker MP may shorten the time until full enteral nutrition is reached.^15^, ^16^ However, data is unclear, and the publications mentioned above by Burchard et al. and Deshmuk et al. could not find a positive association between an enhanced MP and earlier full enteral feeds.^12^, ^14^

This study aimed to analyze the safety and effectiveness of standardized minimally invasive hydrostatic low‐volume saline enemas.

## 2 METHODS

The study was conducted as a controlled interventional study at the neonatological department of the Klinikum Kassel, Germany. This study was divided into three study phases. ELBW infants born between January 2019 and June 2020 were eligible for the control phase, in which infants received manually applied enemas with various solution types, materials, and frequencies. In July and August 2020, the new hydrostatic low‐volume saline enema protocol was introduced, staff were educated, and the first enemas were applied according to the new protocol. No data collection was performed to avoid implementation bias. In the intervention phase, carried out between September 2020 and October 2022, the new enemas were performed in a standardized manner. Data collection was prospective. After completing the study, a post‐hoc survey was conducted in the neonatological ward to assess the staff's opinion on the new enema protocol.

Infants born with a birth weight of ≤1000 g or ≤28 weeks of gestation were eligible for the study. For reasons of simplification, they are further referred to as ELBW infants.

To be included, infants had to be born at Klinikum Kassel or be admitted during the first day of life. Hospital stay had to be longer than 24 h, and the infant needed to suffer from a delayed MP (absence of meconium for >24 h during the time in which the infant only passed meconium as the main stool type) at least once in the first 20 days of life. Exclusion occurred in the absence of parental consent, palliation within the first 72 h of life, in the presence of a genetic disorder, congenital bowel malformation, or intrauterine GI complication.

In the control group, physicians performed all enemas manually, with normal saline of varying volume. The catheter was inserted as deeply as possible. The timing of the first enema application and the frequency were individually chosen based on physicians' assessment.

**Figure 1:**
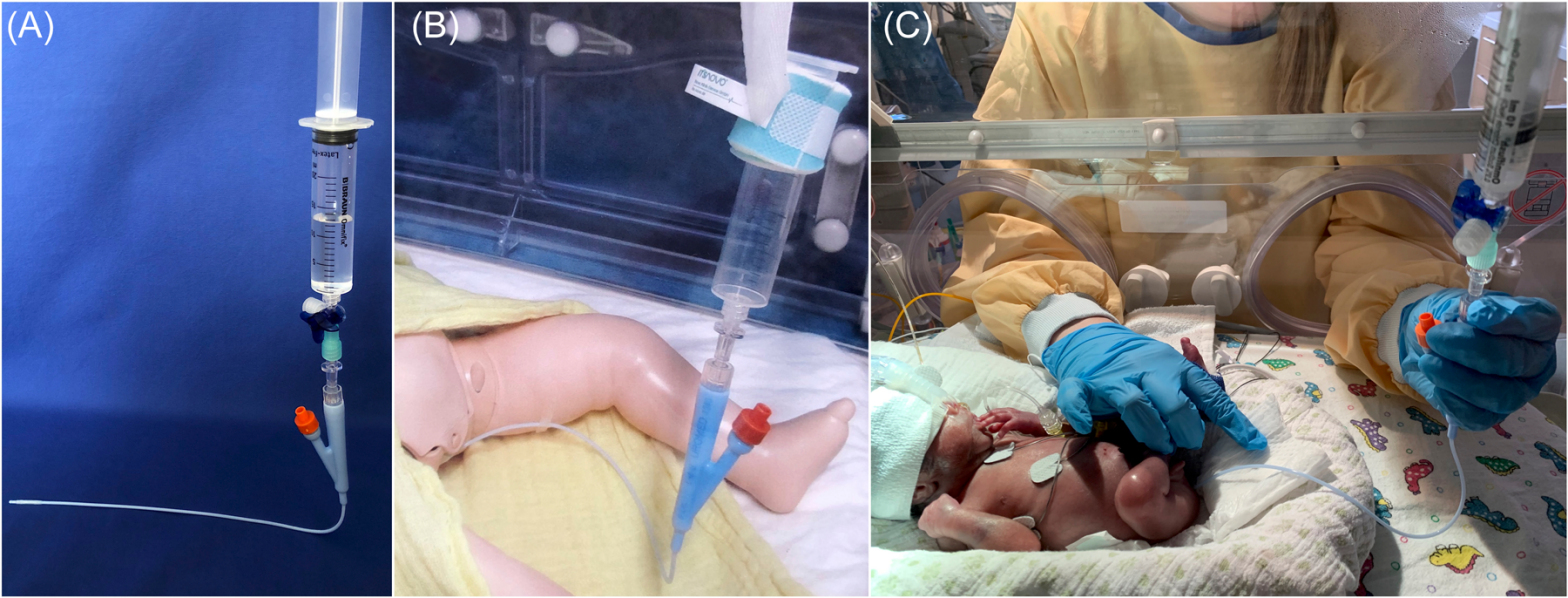
(A) Picture demonstrating the hydrostatic enema system ready for application. Assembly of the urinary catheter, cortical connector, 3‐way stopcock, and syringe, filled with NaCl 0.9%. (B) Picture of enema administration. The enema is demonstrated on a term infant doll. (C) Picture of enema administration. The enema is performed on an extremely low birth weight infant on its seventh day of life (birth weight: 730 g, gestational age at birth 24 + 6).

The new enema protocol, performed in the intervention group, was designed to be as minimally invasive as possible. A urinary catheter (Ch 6) was attached to a 3‐way stopcock via a cortical connector (Figure 1A). On the other side of the 3‐way stopcock, a syringe was connected without its plunger. A total of 10 mL/kg NaCl 0.9% was poured into the syringe, and the plunger was gently pressed onto the syringe to secure the fluid. The prepared enema system was fixed to the incubator at a height of 15 cm to achieve a pressure of 15 cmH_2_O. The catheter tip was lubricated and rectally applied with a depth of 2.5 cm (Figure 1B). The unblocked catheter was secured to the infant's thigh by adhesive strips. The 3‐way stopcock was opened, and the plunger was removed to allow the solution fluid to run through (Figure 1C). An enema was only applied in the absence of bowel passage for more than 24 h or if the last enema was applied more than 24 h ago. Only one enema was performed within 24 h. In contrast to the study by Nakaoka et al.,^17^ no ultrasound was applied to assess whether the terminal ileum was reached.

The feeding regimen was consistent across groups, beginning with an initial oral intake of approximately 20 mL/kg/day. On the first day of life, infants received 12 oral feedings, ideally of mother's milk; if unavailable, human donor milk or Aptamil Prematil HA® was provided. Parenteral nutrition was administered at 80–100 mL/kg/day on Day 1 and adjusted daily based on oral intake. Once infants achieved a minimum oral intake of 80 mL/kg/day, feeds were fortified with 4% fortifier Frauenmilchsupplement. Daily intake was gradually increased by 15–30 mL/kg, according to the protocol and depending on tolerance, until reaching a target volume of 140–160 mL/kg/day, providing an enteral calorie intake of approximately 120–150 kcal/kg/day. Doctors and nurses assessed and adjusted the type and volume of both oral and parenteral nutrition daily during rounds to meet each infant's needs.

The primary outcome was the protocol's safety, assessed by the rate of GI complications (NEC, FIP, MPS), morbidity, and mortality. NEC was diagnosed at a Bell stage of ≥IIA.^17^, ^18^ FIP was diagnosed according to radiological and histological findings, but mostly intraoperative findings. The diagnosis of MPS was made in the absence of stool passage for ≥72 h.

For the secondary outcome, the protocol's effectiveness, several stool and nutritional parameters were assessed at six points in time: Days 2, 4, 6, 10, 15, and 20 of life. The first and last MPs (last thick, black sticky stool, no transitional stool) and the total number of stool passages were evaluated. To assess the nutritional outcome, the number of patients having reached full enteral nutrition (120 mL/kg/day), the need for parenteral nutrition, and the last day of parenteral nutrition were looked at.

SPSS, Version 27.0.1.0 (IBM Corp., Armonk, NY, USA) was used for statistical analysis. An intention‐to‐treat approach was followed to represent usual clinical practice. Besides descriptive statistics, group variances were evaluated by the following tests, according to the data type and distribution: Two‐sample, two‐sided *t*‐test, Mann–Whitney *U*‐test, Chi‐square test, and One‐sided Fisher's exact test. *p* < 0.05 was regarded as a statistically significant result.

For the post hoc survey, the satisfaction, effectiveness, and handling of the new enema system, the child's well‐being during the enema application, and the wish to continue with the new enema system were assessed.

### 2.1 Ethics Statement

This study was conducted in accordance with the ethical principles outlined in the Declaration of Helsinki and adhered to all relevant national and international ethical guidelines for research involving human participants. The study protocol was reviewed and approved by the Ethics Committee of the Landesärztekammer Hessen (State Medical Association of Hesse) on March 26, 2020. The study was registered in the German Register of Clinical Trials (DRKS) under the registration number DRKS00024191. Informed consent was obtained from all participants in accordance with ethical guidelines and institutional review board approval.

## 3 RESULTS

**Figure 2:**
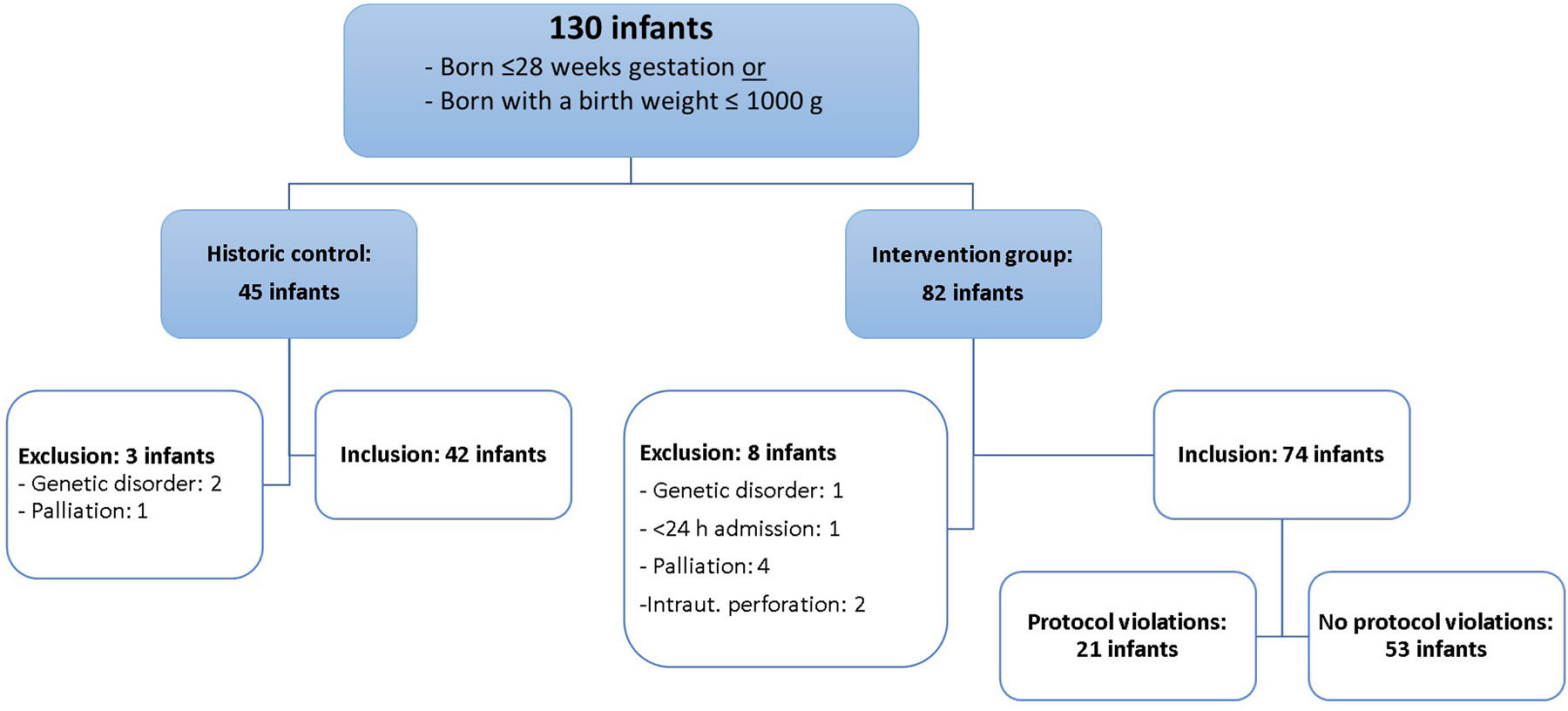
Participant flow. Intraut. perforations, Intrauterine perforations.

One hundred and thirty patients were initially eligible to participate in the study: 45 infants in the control, 3 in the implementation, and 82 in the intervention period. After applying in‐ and exclusion criteria, 42 infants were enrolled in the control, and 74 patients were in the intervention group (Figure 2). Protocol violations occurred in 21 (28.4%) infants in the intervention group, defined as one or more wrongly performed enemas.

### 3.1 Epidemiological characteristics

The demographic characteristics are shown in Table 1. The gestational age at birth was similar (c: 27 + 4 weeks [SD: 1.8] vs. i: 27 + 2 [SD: 2]). The birth weight in the intervention group was significantly lower (*p* = 0.016) compared to the control group, with children weighing only 870 g (range: 340–1370; IQR: 305) compared to 957.5 g (range: 480–1300; IQR: 317.5). Four more characteristics showed statistically significant group variances: Three reasons for delivery (preterm premature rupture of membranes, HELLP syndrome, and other reasons) and APGAR at 5 min (Table 1)

**Table 1:** The study population's demographic data.

	Control group (*N* = 42)	Intervention group (*N* = 74)	*p*‐values
Gestational age at birth; mean (weeks)	193 days (12.43) 95% CI: 188.84–196.59 27 + 4 weeks (1.77) 95% CI: 27 + 0 to 28 + 0	191 days (13.88) 95% CI: 187.66–194.05 27 + 2 weeks (1.98) 95% CI: 26 + 6 to 27 + 5	0.463
Birth weight; median (g)	957.5 (480–1300; 317.5) 95% CI: 900–990	870 (340–1370; 305) 95% CI: 745–920	**0.016**
Gender (male)	19 (45.2%)	39 (52.7%)	0.440
Age at admission (h)	0	0 (0–2, 0)	0.451
Small for gestational age	9 (21.4%)	23 (31.1%)	0.264
Reasons for delivery			
Placental insufficiency	3 (7.1%)	9 (12.2%)	0.303
Pre‐eclampsia/eclampsia	8 (19%)	9 (12.2%)	0.314
HELLP syndrome	1 (2.4%)	11 (14.9%)	**0.029**
PPROM	25 (59.5%)	22 (29.7%)	**0.002**
Preterm labor	27 (64.3%)	40 (54.1%)	0.284
Amniotic infection syndrome	5 (11.9%)	10 (13.5%)	0.804
Other reasons	13 (31%)	43 (58.1%)	**0.005**
Cesarean section	40 (95.2%)	71 (94.6%)	0.624
Apgar score at 1 min	6.5 (1–9; 3) 95% CI: 5–7	5 (1–9; 4) 95% CI: 4–6	0.1
Apgar score at 5 min	8 (5–10; 2) 95% CI: 8–9	7 (1–10; 3) 95% CI: 7–8	**0.05**
Apgar score at 10 min	9 (7–10; 2) 95% CI: 9–10	9 (4–10; 2) 95% CI: 9–9	0.309
Multiple birth	16 (38.1%)	25 (33.8%)	0.641
Mother's age	29.5 (19–41; 12) 95% CI: 28–33	31.5 (18–48; 10) 95% CI: 29–33	0.603
Tocolysis	27 (64.3%)	46 (62.2%)	0.82
Antenatal corticosteroids	39 (92.86%)	63 (85.1%)	0.22
Medical diagnoses			
Bronchopulmonary dysplasia	13 (31%)	25 (33.8%)	0.755
Ventricular hemorrhage	7 (16.7%)	12 (16.2%)	0.95
Retinopathy of prematurity	19 (45.2%)	27 (36.5%)	0.354
Persistent ductus arteriosus	15 (35.7%)	35 (47.3%)	0.226
Catheter‐associated infections	7 (16.7%)	14 (18.9%)	0.762
Inguinal hernia	3 (7.1%)	15 (20.3%)	0.061
Duration of intubation (h)	17 (0–418; 95.25) 95% CI: 8.33–47.83	16.5 (0–472.25; 96.67) 95% CI: 2.66–46.92	0.447
Length of hospital stay (days)	71 (35–160; 23) 95% CI: 61–79	81 (25–246; 343) 95% CI: 73–86.99	0.191
Mortality	3 (7.1%)	2 (2.7%)	0.25

*Note*: Figures are represented in frequencies (%), mean (standard deviation), and median (minimum–maximum; interquartile range), respectively. Confidence intervals are given for continuous data. Significant results are represented in bold font. Patients who died in the hospital were not included for the duration of intubation and the length of hospital stay.

Abbreviations: HELLP, Hemolysis Elevated Liver enzymes Low Platelet count; *N*, number of participants; PPROM, preterm premature rupture of membranes.

The clinical data displayed various group differences. Whereas the median oxygen saturation on the fourth day of life was 96% (range: 91.5–99.6; IQR: 4.1) in the control group, it was only 93.9% (range: 89.3–99.3; IQR: 3.6) in the intervention group, with *p* = 0.019. The FiO2 and the general respiratory status, divided into unsupported breathing, noninvasive ventilation, and invasive ventilation, were similar on that day and on any other measuring points. Only on Day 20 of life, 37.5% (*N* = 15) of infants in the control group were able to breathe unsupported, which was only the case for 18.1% (*N* = 13) in the intervention group (*p* = 0.02).

More infants in the intervention group needed antibiotic treatment on Days 4, 6, and 10 of life: Day 4 (c: 56.1%, *N* = 23 vs. i: 78.1%, *N* = 57; *p* = 0.014), Day 6 (c: 36.6%, *N* = 15 vs. i: 65.8%, *N* = 48; *p* = 0.003), and Day 10 (i: 12.2%, *N* = 5 vs. 36.1%, *N* = 26; *p* = 0.006). As a result of the reduced birth weight in the intervention group, the absolute weight results were always significantly lower in the intervention group. However, a significantly higher weight gain was observed in the second week of life in the intervention group, with 122.7 g (SD: 62.4) compared to 89.9 g (SD: 65.7) in the control group (*p* = 0.01).

In the control group, ≥1 enema was applied in 90.5% (*N* = 38) of infants, and 97.3% (*N* = 2) of infants in the intervention group. However, significantly fewer enemas were performed per child in the intervention group, 4 (range: 0–33, IQR: 5) compared to 8 (range: 0–55, IQR: 8) in the control group (*p* = 0.04).

### 3.2 Improved enteral feeding tolerance in the intervention group despite lower stool frequency

The first MP was comparable between the groups and occurred after a median of 19.2 h (range: 0.2–91.2; IQR: 22.9) in the control group and 20.3 h (range: 0–91.7; IQR: 26.5) in the intervention group (*p* = 0.943). However, the last MP occurred significantly later in the intervention group, after 7 days (range: 2–19, IQR: 4) compared to 6 days (range: 2–13; IQR: 3) in the control group, with *p* = 0.019. Moreover, significantly fewer stools were passed in the intervention group in the first 20 days of life (*p* = 0.002). Meanwhile, infants in the intervention group excreted 61.9 stools on average (SD: 15.8), while infants in the control group had 72.2 stool passages (SD: 13.5). Interestingly, feeding tolerance was significantly better in the intervention group, with significantly more infants on full enteral nutrition on Day 10 of life, 31.5% (*n* = 23), compared to 14.6% (*n* = 6) in the control group (*p* = 0.047).

Parenteral calorie intake differed between the groups, with the intervention group showing higher intake at the first four measurement points (Days 2, 4, and 6: *p* < 0.001; Day 10: *p* = 0.003). However, the percentage of infants requiring parenteral nutrition and the volume of intravenous fluids needed were comparable between groups across all six measurement points. The duration of parenteral nutrition was also similar: in the control group, the median duration was 14 days (range: 8–88 days; IQR: 10), while in the intervention group, it was 16 days (range: 6–246 days; IQR: 14), with no statistically significant difference (*p* = 0.105).

### 3.3 A trend towards lower morbidity and incidence of GI complications in the intervention group

**Figure 3:**
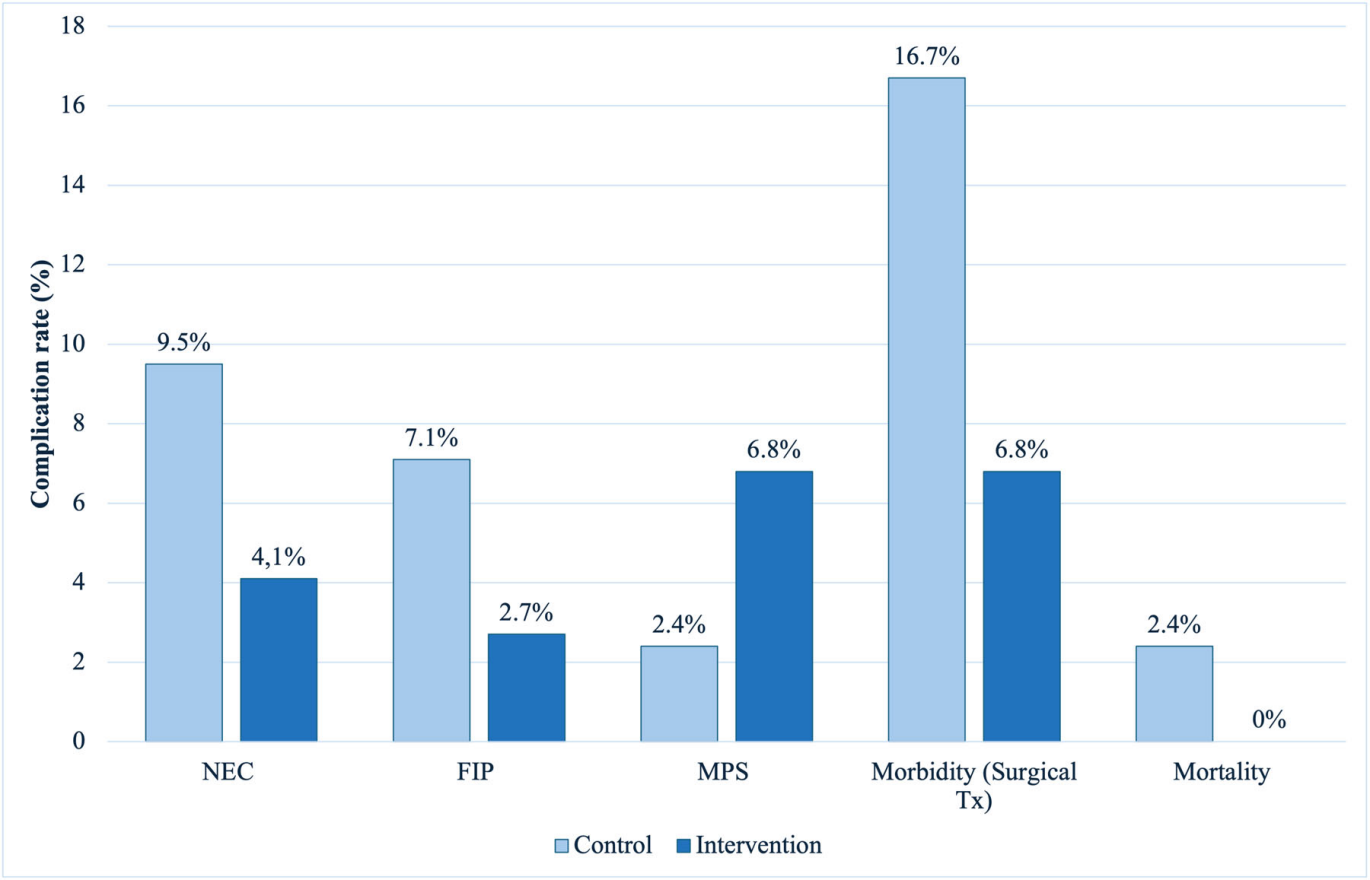
The study population's primary outcome measures. The bar chart illustrates the gastrointestinal complication, morbidity, and mortality rates between the control and the intervention group. No significant differences were found between the groups. Although a trend toward reduced morbidity could be observed in the intervention group (6.8% vs. 16.7%; *p* = 0.08). FIP, focal intestinal perforation; MPS, meconium plug syndrome; NEC, necrotizing enterocolitis; Tx, therapy.

There were no significant differences in the primary outcome data (Figure 3). 4.1% of patients in the intervention group developed NEC compared to 9.5% in the control group (*p* = 0.214), 2.7% had FIP compared to 7.1% (*p* = 0.25), and MPS occurred in 6.8% compared to 2.4% (*p* = 0.29). Although not significant (*p* = 0.088), there was a trend for reduced intestinal morbidity in the intervention group, with only 6.8% (*N* = 5) of infants needing surgery compared to 16.7% (*N* = 7) in the control group. Mortality rates were similar (c: 2.4% vs. i: 0%; *p* = 0.362). However, combined mortality and morbidity were significantly lower in the intervention group (6.8% vs. 19.1%, *p* = 0.46).

Eleven of 28 (39.3%) eligible nursing staff members participated in the post‐hoc survey. Three members (27.3%) rated the new enema protocol as more comfortable, and the remaining eight healthcare providers (72.7%) rated it slightly more comfortable.

## 4 DISCUSSION

This interventional control study analyzed the safety and efficacy of a standardized minimally invasive hydrostatic low‐volume enema protocol. Whereas we found no significant differences in the primary outcome data, we noted a clear trend towards a decreased intestinal morbidity in the intervention group and a significantly better enteral feeding tolerance in the intervention group. When comparing our baseline surgical NEC and FIP rate with nationwide data, the base rate in our control group was higher at 16.1% versus 7.0%.^18^ However, the overall mortality was substantially lower at 7.1% compared to 16.5% for infants between 500 and 999 g. Thus, the combined mortality and morbidity were quite similar in our unit compared to the nationwide data, with 23.1% versus 23.6%.^18^ Thus, the differences in surgical NEC and FIP rates may reflect our unit's local ethical management policies. More recent nationwide German data does not report specific incidences for infants between 500 and 999 g.^19^

Notably, if we look at the intervention group, the morbidity from surgical NEC and FIP dropped to 6.8%, similar to the nationwide data. In fact, with an overall mortality rate of 2.4% in the intervention group, the combined mortality and morbidity were only 9.2% compared to 23.6% as reported by Jeschke et al.^18^ Even though the observed differences might just be statistical noise, it is of note that the intervention group in our study had a significant lower birth weight, less favorable respiratory parameters, more antibiotic and steroid treatment at the beginning of life, and more patients being delivered because of HELLP syndrome and was thus even more prone to GI complications.^4^, ^7^, ^20^, ^21^, ^22^, ^23^, ^24^, ^25^, ^26^ Considering these factors, the trend toward a reduced morbidity and the significantly lower combined mortality and morbidity in the intervention group might be more significant than the actual data show.

Regarding MPS, it is difficult to find data for rates in the literature since the disease is ill‐defined.^8^, ^27^ In 2020, a study conducted on 1543 infants with very low birth weight found a rate of meconium‐related ileus in 4.47% of infants.^27^ Since we only analyzed ELBW infants in our study, it is understandable why our population produced a slightly higher MPS rate of 6.8%. A delayed last meconium evacuation and a reduced number of stool passages could be observed in the intervention group. This might have been caused by the fewer enemas applied in the intervention group (4 vs. 8), resulting from the pre‐set time intervals of the protocol. However, the literature on this is contradictory. As mentioned above, the meta‐analysis by Burchard et al. saw a correlation between rectal interventions and an enhanced full meconium evacuation. However, no clinical benefit was found since NEC, mortality, and enteral feeding were not altered.^14^

Protocol violations occurred in 28.4% of infants in the intervention group, a rate comparable to similar studies in this field, which report protocol violation rates of 28.4% and 24.4%, respectively.^28^, ^29^ These findings suggest that achieving complete adherence to new protocols remains challenging in clinical practice.

Parenteral calorie intake was initially higher in the intervention group, likely due to the significantly lower average birth weight within this group. However, overall requirements for parenteral nutrition, intravenous fluid volumes, and duration of parenteral nutrition were comparable between groups.

Importantly, in this study, more infants in the intervention group had reached full enteral nutrition on Day 10, compared to the control group, despite the later MP and lower number of rectal enemas. This clearly demonstrates that other factors are more important for feeding tolerance than MP. This is also supported by three recent publications that failed to establish a positive association between rectal interventions and nutritional outcomes.^11^, ^12^, ^14^ Interestingly, two studies looked at stool passage and nutrition in the absence of rectal interventions, showing that quicker normalization of stool passage is correlated with an improved enteral nutritional intake.^5^, ^16^ Therefore, it is likely that a timely MP in the absence of rectal interventions indicates a more mature GI tract. However, a forced enhanced MP by rectal interventions does not seem to alter nutritional outcomes. Hence, a delayed MP is likely a symptom of a more immature GI tract. It is not sensible to influence it in a forced manner by intensive interventional methods.

It is important to note that this study is a single‐center study, with data for the control group collected retrospectively. Therefore, larger prospective randomized controlled trials are needed to validate these findings.

## 5 CONCLUSION

Hydrostatic low‐volume enemas show a good safety profile, no increase in GI complications, a trend towards reduced morbidity, and more infants have reached full enteral nutrition on Day 10 of life. Further, the minimally invasive application form improved the patients' well‐being during enema administration.

## CONFLICT OF INTEREST STATEMENT

The authors declare no conflicts of interest.
